# Revisiting Treatment Strategies: Addressing Epithelial-to-Mesenchymal Transition-Induced Resistance in Hepatocellular Carcinoma

**DOI:** 10.34133/bmef.0144

**Published:** 2025-06-24

**Authors:** Roghayeh Naserkhaki, Bahare Shokouhian, Yaser Tahamtani, Arezoo Khosravi, Siavash Iravani, Ali Zarrabi, Massoud Vosough

**Affiliations:** ^1^ Department of Developmental Biology, University of Science and Culture, Tehran 1665658822, Iran.; ^2^ Department of Regenerative Medicine, Cell Science Research Center, Royan Institute for Stem Cell Biology and Technology, ACECR, Tehran 1665659911, Iran.; ^3^ Department of Stem Cells and Developmental Biology, Cell Science Research Center, Royan Institute for Stem Cell Biology and Technology, ACECR, Tehran, Iran.; ^4^ Department of Basic and Population Based Studies in NCD, Reproductive Epidemiology Research Center, Royan Institute, ACECR, Tehran, Iran.; ^5^ Department of Genetics and Bioengineering, Faculty of Engineering and Natural Sciences, Istanbul Okan University, Istanbul 34959, Turkiye.; ^6^ Independent Researcher, Isfahan, Iran.; ^7^ Department of Biomedical Engineering, Faculty of Engineering and Natural Sciences, Istinye University, Istanbul 34396, Turkiye.; ^8^ Graduate School of Biotechnology and Bioengineering, Yuan Ze University, Taoyuan 320315, Taiwan.; ^9^ Department of Research Analytics, Saveetha Dental College and Hospitals, Saveetha Institute of Medical and Technical Sciences, Saveetha University, Chennai 600 077, India.; ^10^ Experimental Cancer Medicine, Institution for Laboratory Medicine, Karolinska Institutet and Karolinska University Hospital-Huddinge, Huddinge, Sweden.

## Abstract

One of the major therapeutic challenges for hepatocellular carcinoma (HCC), the most form of primary liver cancer, is how to overcome drug resistance. Due to the high failure rate of systemic therapy in treating advanced HCC patients and the increasing recurrence rate, HCC is a highly lethal malignancy. Primary and acquired drug resistance are major contributing factors to the patients with advanced HCC who do not respond effectively to long-term systemic therapy. Therefore, it is essential to look into the molecular processes that lead to drug resistance. Different studies have indicated that epithelial-to-mesenchymal transition (EMT) plays a critical part in the emergence of drug resistance. Several signaling pathways regulate this phenomenon. This review primarily concentrates on drug resistance triggered by EMT, especially in the context of HCC. The key signaling pathways that cause drug resistance in HCC, including transforming growth factor-β and epidermal growth factor receptor signaling, liver cancer stem cells, and noncoding RNAs, are highlighted in the present study, along with the most recent molecular targets discovered to prevent drug resistance. These targets could help develop novel and combinatory HCC therapy approaches. Therefore, this review aims to provide both the latest findings on molecular basis and potential solutions for HCC drug resistance.

## Introduction

Among primary liver cancers, hepatocellular carcinoma (HCC) is the most prevalent type [1], which has the fourth leading cause of cancer-related mortality worldwide [2]. It is estimated that by 2045, the global liver cancer death rate would be around 71.6% [3]. Further, HCC has a relative 5-year survival rate of only 18%, which emphasizes its poor prognosis [4]. The risk factors for HCC encompass chronic infection with hepatitis B (HBV) and hepatitis C (HCV) viruses, alcohol addiction, nonalcoholic fatty liver disease, diabetes, obesity, tobacco use, hereditary hemochromatosis, and contact with dietary contaminants like aflatoxins and aristolochic acid [5–7]. Current therapeutic approaches encompass surgical resection, liver transplantation radiofrequency ablation, transarterial chemoembolization, microwave ablation, and rarely systemic chemotherapy [8]. HCC patients frequently have treatment resistance, especially those with advanced or metastatic cancer, even if these treatments initially aid in treating the illness [9,10]. There are different mechanisms of chemoresistance that cause the high relapses after chemotherapy and targeted molecular therapy of HCC. These mechanisms include drug uptake and export, drug metabolism, changes in drug targets, balance between pro-survival and pro-apoptotic factors, DNA repairing, adaptation to tumor microenvironment, and phenotypic transition. Epithelial-to-mesenchymal transition (EMT) is one of the important mechanisms that cause chemoresistance in HCC [11,12]. Mesenchymal phenotype that epithelial cells acquire during EMT change their ability to migrate and invade, produce cancer stem cells (CSCs), promote metastasis, and cause resistance to treatment [13]. This review presents the mechanisms of EMT that cause drug resistance in HCC, helping to understand the generation of treatment resistance, and contains potential EMT-targeted therapies for HCC.

## EMT

The EMT is a well-preserved cellular process in which stationary, polarized epithelial cells shed their cell polarity and intercellular adhesion, transforming into motile mesenchymal cells with enhanced migratory and invasive capabilities. Importantly, the mesenchymal-to-epithelial transition (MET) mechanism allows mesenchymal cells to return to an epithelial phenotype. This indicates that EMT is not irreversible [14–16]. EMT takes place across various physiological and pathological contexts, such as embryonic development, wound repair, tissue fibrosis, and cancer progression [17]. In cancer pathogenesis, EMT is associated with initiation and progression of tumor, development of resistance to conventional therapies, and metastasis [14]. A dynamic process known as epithelial–mesenchymal plasticity (EMP) includes the transformation of epithelial cells into mesenchymal cells and vice versa. Gene expression alterations, decreased cell–cell adhesion, and increased migratory and invasive capabilities are characteristics of this transition. EMP is crucial for normal physiological functions including wound healing and embryonic development, but it also plays a major role in the progression and metastasis of cancer. Cancer cells’ fitness is enhanced by EMP both during tumor growth and in response to therapies. Several studies indicate that dynamic changes in epithelial/mesenchymal states lead to the formation of heterogeneous tumor cell populations with different drug sensitivities. Recent studies indicate that hybrid epithelial/mesenchymal tumor cells have a remarkable correlation with resistance to therapy, presumably as a result of their enhanced capacity to survive a variety of therapeutic stressors [18–20].

### Microenvironment drivers of the EMT

**Fig. 1.**
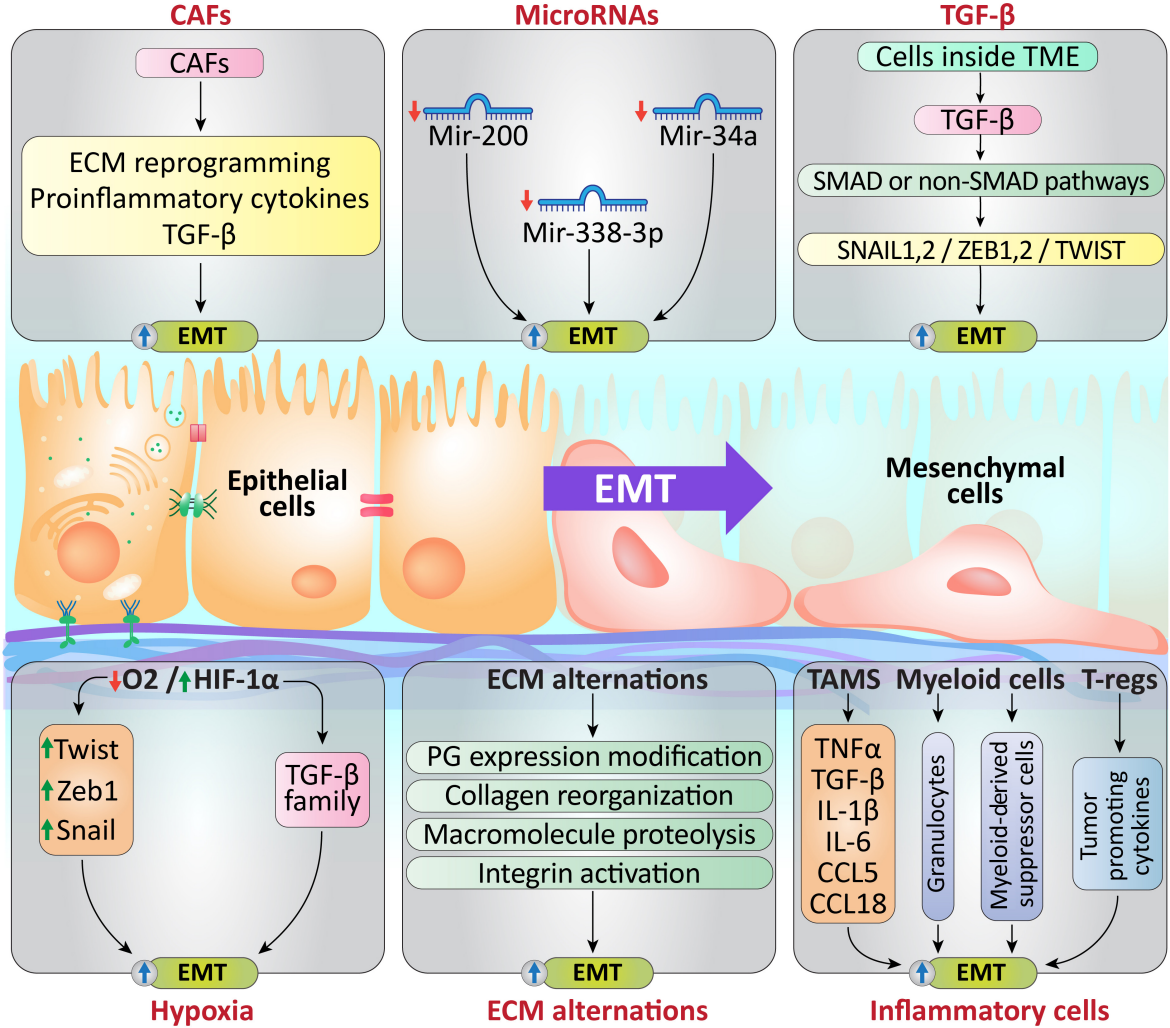
Microenvironment drivers of the EMT. The development of therapy resistance is significantly impacted by EMT drivers related to the tumor microenvironment, including cancer-associated fibroblasts (CAFs), tumor-associated macrophages (TAMs), changes in the microRNAs, hypoxic conditions, extracellular matrix (ECM), and inflammatory and immune cells. These factors trigger various signaling pathways, including TGF-β, TNF-α, TGF-β, IL-1β, IL-6, HIFs, NOTCH, and WNT, which induce EMT-related transcription factors.

Cancer-associated fibroblasts (CAFs), tumor-associated macrophages (TAMs), any changes in the content of extracellular matrix (ECM), hypoxia, and immune and inflammatory cells are examples of EMT-related variables in the tumor microenvironment. These factors have remarkable important impact on the development of therapy resistance and could be potential therapeutic targets in future studies [14,21]. Figure 1 summarizes microenvironment drivers of the EMT and their related mechanisms.

Hypoxia and induction of hypoxia-inducible factor-1α (HIF-1α) are important microenvironmental factors that up-regulate several EMT regulators, including Snail, Twist, and Zeb1. By directly interacting with the hypoxia-response element (HRE) in the TWIST proximal promoter, HIF-1 controls the expression of TWIST. EMT and metastatic characteristics were reversed by small interfering RNA (siRNA)-mediated suppression of TWIST in HIF-1 overexpressing or hypoxic cells [22,23]. HIF-1 may have an effect by interacting with other variables. For example, HIF-1 interacts with components of the transforming growth factor (TGF) family, which are also powerful inducers of EMT [24].

The ECM, a 3D structure surrounding cells in a distinct environment, serves as a vital part of the tumor microenvironment, offering both physical and biochemical support to the cells [25]. ECM structural and functional alterations, including proteoglycan expression modifications, collagen interactome reorganization, macromolecule proteolysis, and integrin activations, drive EMT [26,27]. The majority of available treatments targeting EMT focus on blocking upstream inducers of EMT. Several studies have shown that it is possible to target the ECM, several ECM stiffness regulators, different mechanosensors, and mechanotransducers. Several inhibitors targeting CD44, DDR, LOX/LOX2, integrins, and FAK have been established, and in preclinical studies, some of these inhibitors have revealed anticancer properties [21,28–32].

EMT can be triggered and maintained by inflammatory cells and their secretome [33,34]. Within the tumor microenvironment, TAMs comprise the biggest group of nontumor cells [35]. TAMs are macrophages that reside in the tumor microenvironment [36,37]. TAMs release pro-inflammatory cytokines like tumor necrosis factor-α (TNF-α), TGF-β, interleukin-1β (IL-1β), IL-6, CCL5, CCL18, and CXCL13, playing a critical role in both induction and sustaining EMT. TAMs use a variety of modes of action to activate the EMT process [38–41]. Granulocytes and myeloid-derived suppressor cells, among other myeloid cell types, can also initiate EMT [42–44]. The activation of EMT is also mediated by lymphocytes that infiltrate tumors, such as regulatory T cells, mainly by releasing cytokines that promote inflammation and other tumor-promoting cytokines [45].

CAFs, myeloid-derived cells (mostly macrophages), and endothelial cells are the most common nontumor cells in the tumor milieu [46]. By promoting EMT-related processes such as remodeling the ECM, altering tumor phenotype, and influencing other cells within the tumor microenvironment, CAFs drive cancer progression. These activities are enabled by soluble factors and cellular connections between CAFs and other stromal cells or tumor cells [47–49]. TGF-β and pro-inflammatory cytokines are examples of EMT-enhancing factors generated by CAFs [48,50]. Further, multiple studies reveal that there is potential to treat cancer with therapeutic targeting of CAF-stimulated EMT. Several drugs have been demonstrated to inhibit CAF-stimulated EMT by impairing the IL-6/IL-6R signaling, such as cucurbitacin I (JSI–124), tocilizumab, and siltuximab [51–53].

EMT is also controlled by microRNAs (miRNAs). These substances either down-regulate EMT-related transcription factors or, conversely, operate as working communicators between them to regulate the process of EMT [54]. A set of 30 miRNAs, along with a group of target genes, has been identified as key regulators of the interactions among TGF-β, Notch, and Wnt signaling pathways during EMT [55]. MiRNAs like miR-200, miR-34, and miR-338-3p, among others, have been shown to play significant roles in governing EMT [56–58].

EMT is also mediated by TGF-β [59], which is a multifunctional cytokine formed by various cells within the tumor microenvironment and is a key inducer of EMT, immune evasion, and metastasis during cancer progression [60]. In the initial phases of tumor development, TGF-β usually inhibits tumor growth. However, in advanced phases, it promotes malignancy, especially by acting as the main inducer of EMT, which leads to the tumor progressing toward metastasis and developing resistance to chemotherapy. Numerous EMT transcription factors, such as SNAIL1, SNAIL2, ZEB1/2, and TWIST, can be induced by TGF-β through SMAD or non-SMAD signaling pathways [59,61,62]. Recent studies have shown that in tumor-bearing mice, YM101, a new bispecific antibody that targets TGF-β and PD-L1 concurrently, can restore immunosuppression and prevent EMT [63].

### Mechanisms of EMT-induced drug resistance

Multidrug resistance (MDR) in the context of cancer treatment is cancer cells’ capacity to withstand the exposure to multiple types of anticancer drugs [64]. Evaluating the drug response of cancer cells before and after induction of EMT is a simple method to examine the basic association between drug resistance and EMT. In this context, several studies prove this potential connection and imply that EMT can cause cancer cells to become drug-resistant [65]. Growing evidence indicates that EMT signaling has a role in chemoresistance, immune suppression, and cancer stemness, which can lead to tumor metastasis [14,66–69]. The potential molecular pathways involved in the connection between EMT and medication resistance are covered in the following section.

#### MDR-associated molecules

**Fig. 2.**
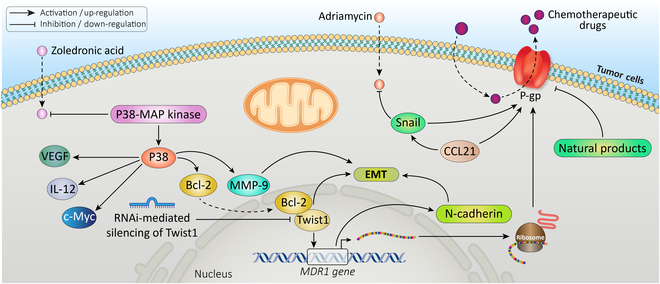
MDR-associated molecules cause EMT-induced drug resistance. Studies indicate that different factors such as RNAi-mediated silencing of Twist1, Snail overexpression, CCL21 cytokine, natural products, BCL-2 protein, and P38 affect P-gp and EMT-induced drug resistance.

The drug response of cancer cells is directly influenced by effector molecules, including P-gp (P-glycoprotein) and BCL-2. The contribution of EMT to drug resistance would be supported by studies indicating its relationship with these direct effectors (Fig. 2) [65,70]. The expression of the multidrug transporter P-gp, an energy-dependent drug efflux pump, is the primary mechanism driving MDR in cultured cancer cells [71,72]. P-gp, produced in humans by the *MDR1* gene, was among the earliest identified members of the extensive adenosine triphosphate (ATP)-binding cassette (ABC) transporter family, known for its ATP-dependent transport functions [73,74]. Dysfunction of ABC transporters is a key contributor to MDR [75]. P-gp enhances MDR by pumping out chemotherapeutic drugs and decreasing their intracellular concentration. Thus far, P-gp overexpression has been identified in several chemoresistant cancer types [76].

Studies showed that the RNA interference (RNAi)-mediated silencing of Twist1 decreased P-gp levels in HeLa cervical cancer cells, hindered cell efflux function, and made the cells more sensitive to cisplatin therapy [77]. Besides, studies of human colorectal cancer cells indicated that the transcriptional factor Twist drives both EMT and the expression of P-gp [78]. Additionally, Snail overexpression in MCF-7 cells results in adriamycin resistance and a significant increase in P-gp [79]. Moreover, treatment of colorectal cancer HCT116 cells with the CCL21 cytokine, which promotes chemoresistance, increased P-gp expression and efflux function in addition to inducing Snail overexpression [80]. Moreover, research on cisplatin-resistant human oral squamous cell carcinoma cell lines revealed that *MDR1* overexpression, an increase in P-gp functional activity, increased expression of EMT-related markers like N-cadherin. This overexpression resulted in increased cell migration [81]. Recently, several studies have demonstrated that miRNAs induce cancer EMT and P-gp up-regulation and imply that miRNAs that regulate EMT contribute to P-gp alteration [70]. Since overexpression of P-gp has been observed in a number of chemoresistant types of cancer, several mechanisms have been proposed to inhibit P-gp-related MDR. These mechanisms include (a) reducing P-gp efflux activity by changing its conformation or preventing P-gp-chemotherapeutic drug binding, (b) inhibiting P-gp expression to reduce efflux, and (c) knocking out the ABCB1 gene. Natural products, synthetic compounds, and biological techniques are examples of potential strategies that can inhibit P-gp [76,82–88].

In addition to P-gp, BCL-2 family proteins are also connected with EMT. According to multiple studies, the expression of the BCL-2 protein could inhibit apoptosis and contribute to MDR development. Studies suggested that BCL-2 family proteins may have specific functions in EMT, and some pathways might co-regulate EMT and BCL-2 family proteins [65,89,90]. For instance, let-7c was shown to be down-regulated in lung adenocarcinoma cells, which led to the development of docetaxel resistance. Conversely, let-7c up-regulation reversed the EMT phenotype of the cells and suppressed endogenous Bcl-xL [91]. Additionally, Bcl-xL overexpression has also been linked to breast cancer metastasis through EMT induction [92].

In prostate cancer cells, P38–mitogen-activated protein kinase (MAPK) activation is a key factor in inducing zoledronic acid resistance and developing an aggressive and invasive phenotype. Zoledronic acid resistance, EMT marker expression, and invasion are all totally reversed by P38-MAPK inhibitors. Also, P38 inhibitors decrease VEGF, Eotaxin-1, IL-12, matrix metalloproteinase-9 (MMP-9), Bcl-2, and c-Myc expression [93].

In colon cancer cells, increased TLR4 expression after chemotherapy enhances cell survival and EMT through phosphorylation of GSK3β. The inhibition of apoptosis occurs through up-regulation of the expression of anti-apoptosis-related BCL-2 family proteins such as BCL-2, XIAP, and survivin [94]. Recent studies by Xu et al*.* [95] on triple-negative breast cancer (TNBC) indicated that TGF-β has a key function in TNBC epirubicin resistance by regulating stemness, EMT, and apoptosis. Remarkably, epirubicin-resistant MDA-MB-231 cells showed different expression levels of Bcl2, Bax, E-cadherin, N-cadherin, and cyclin D1. In addition, studies on estrogen receptor-positive (ER^+^) breast cancer cells revealed that TGF-β could cause increase in expression of c-Myb, which is necessary for the expression of EMT markers. Using c-Myb siRNAs to transfect adjusted the TGF-β-induced decrease in E-cadherin expression and prevented the rise of Slug and Bcl-2 expression. In the meantime, c-Myb-silenced TGF-β-treated ER^+^ cell lines had an increase in apoptosis caused by etoposide [96]. These findings suggested that c-Myc may be involved in the regulation of *BCL-2* and EMT. Apart from possible co-regulation through alternative molecules and pathways, direct interaction between BCL-2 and proteins linked to EMT has also been identified [65].

Sun et al*.* [97,98] have reported on the structural and functional interactions between Twist1 and Bcl-2. It was found that the basic helix-loop-helix DNA-binding domain of Twist1, along with two distinct regions of the Bcl-2 protein, played a role in the interaction between Bcl-2 and Twist1. The assembly of the Bcl-2/Twist1 complex facilitated the nuclear transport of Twist1 and triggered the transcription of genes linked to enhanced tumor cell plasticity, metastasis, and vasculogenic mimicry. Furthermore, it was found that nuclear expression of Bcl-2 and Twist1 was associated with a low rate of survival in HCC patients, which was also supported by another study.

#### NF-κB

**Fig. 3.**
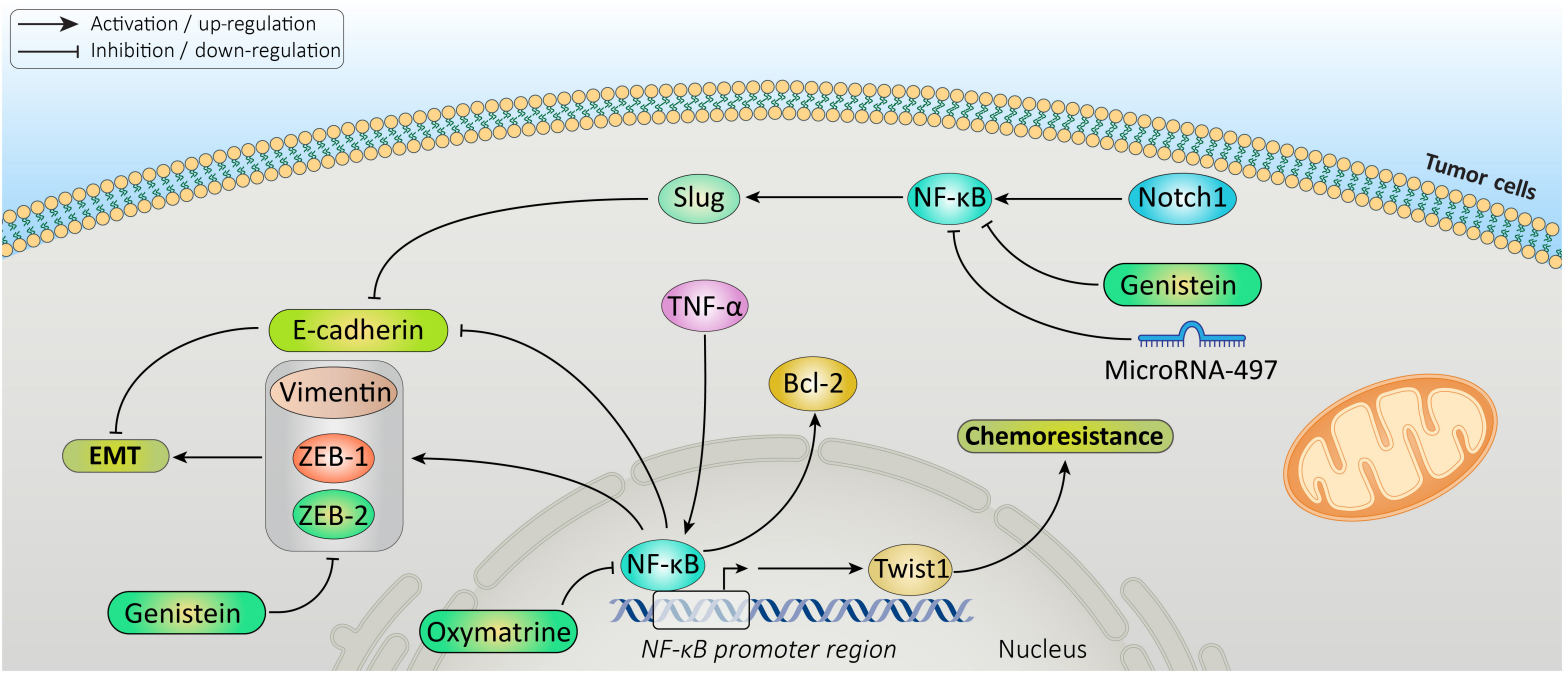
NF-κB causes EMT-induced drug resistance. Different factors such as TNF-α, Notch1, isoflavone genistein, microRNA-497, and oxymatrine affect NF-κB pathway. Induction of NF-κB pathway leads to increase of mesenchymal transcription factors such as vimentin, Zeb1, and Zeb2, which leads to EMT and drug resistance.

Cancer drug resistance may be regulated by nuclear factor κB (NF-κB). After translocating the NF-κB transcription factor into the nucleus, it attaches to a particular promoter region of apoptosis-related molecules. For instance, the expression of Bcl-2 is increased when active NF-κB binds to its promoter region. The NF-κB pathway is linked with drug resistance in various tumors, including gastric, breast, ovarian, colorectal, prostate, and other cancers. NF-κB may also have a role in EMT. Therefore, one of the mechanisms that links EMT to the drug resistance may be the NF-κB pathway (Fig. 3). Various E-cadherin transcriptional repressors are related to NF-κB and EMT [65,99–102]**.**

In breast cancer, the nontransformed mammary epithelial cell line MCF10A displayed typical mesenchymal characteristics, including reduced E-cadherin and enhanced vimentin, ZEB1, and ZEB2, when following transfection with the P65 subunit of NF-κB or exposure to TNF-α (a potent NF-κB inducer). TNF-α withdrawal from the culture medium reversed the EMT-like phenotype associated with decreased ZEB1, ZEB2, and vimentin expression and increased E-cadherin expression [103]. Another study on colon cancer cells indicated that the isoflavone genistein can induce apoptosis and inhibit proliferation by reversing EMT via the Notch1/NF-κB/Slug/E-cadherin pathway. The expression of NF-κB p65 and p-NF-κB p65 was significantly down-regulated by genistein. Genistein elicits an anti-metastasis effect through E-cadherin up-regulation and down-regulation of N-cadherin, FOXC1, FOXC2, ZEB1, ZEB2, Snail2/slug, and TWIST1 [104]. Besides, another study reported that oxymatrine inhibits EMT by reducing the activation of the NF-κB signaling pathway in colon cancer cells [105].

The study of Pham et al*.* [106] on gene chip screening revealed that NF-κB activation increased Twist-1 and inhibited the cellular destruction brought on by chemotherapy. Their study also revealed that the protective activity of Twist-1/2 requires the suppression of inhibitory phosphorylation of Bcl-2 on Ser^87^. Their findings suggest that Twist-1 and Twist-2 are crucial for NF-κB-dependent chemoresistance. Other research also showed that NF-κB was required for Twist-1-mediated EMT and that is combined with in vitro/in vivo models of breast carcinogenesis. NF-κB was required for both formation and maintenance of EMT and in vivo metastasis [107]. Additionally, a recent study used a model of primary breast cancer cells to explore the link between paclitaxel resistance and the mesenchymal phenotype, along with the underlying mechanisms involved. According to this study, mesenchymal primary breast cancer cells exhibited paclitaxel resistance, which was linked to the elevated NF-κB p65 and IKKα/β levels. In the meantime, paclitaxel resistance in mesenchymal breast cancer cells was reversed by inhibiting NF-κB activation [108]. Another recent study discovered that miRNA-497 overexpression prevents gemcitabine resistance, migration, and invasion in pancreatic CSCs by targeting NF-κB1 [109]. These studies suggest that NF-κB could regulate both drug resistance and EMT, and EMT might influence sensitivity to drugs by controlling NF-κB.

#### Cancer stem cells

CSCs, also known as tumor-initiating cells, represent a subgroup of cancer cells capable of self-renewal and differentiation into various cancer cell types when exposed to chemotherapeutic agents. This ability can result in drug resistance and cancer relapse [110–112]. Bonnet and Dick [113] made the initial discovery of CSCs in 1997 when they identified a subset of leukemia cells with CD34^+^/CD38^−^ antigenic phenotypes that could lead to tumor formation in nonobese diabetic/severe combined immunodeficient recipient mice after transplantation. Aside from blood cancer, CSCs have also been found in a number of solid cancers, such as melanoma, brain, lung, liver, pancreatic, colon, breast, and ovarian cancer [114–121]. The origin of CSCs may vary based on the tumor type, with several theories proposing different sources: mutated adult stem cells, altered adult progenitor cells, or differentiated cells that have acquired stem-like properties through a process of dedifferentiation [110]. Multiple cell surface markers, including CD133, CD24, CD44, EpCAM (epithelial cell adhesion molecule), THY1, ABCB5 (ATP-binding cassette B5), and CD200, have been confirmed to identify populations highly enriched in CSCs [114,118,120,122]. The selection of cell surface markers for CSC identification may differ according to the specific traits and phenotypes of each cancer type. For example, liver cancer CSCs are frequently isolated using surface markers like CD133^+^, CD44^+^, CD49f^+^, CD90^+^, ABCG2, CD24^+^, and ESA [123,124].

Numerous studies indicated that most cancer cells may be eradicated by cancer treatment; nevertheless, CSCs are enhanced during chemotherapy and can continue to live, proliferate, and trigger a cancer recurrence with greater resistance against therapy. Indeed, CSCs exhibit treatment resistance across a wide range of cancer types [111,112,125,126]. For instance, CD133^+^ CSCs in glioblastoma displayed resistance to chemotherapy drugs such as temozolomide, carboplatin, paclitaxel, and etoposide [127]. In breast tumors, CSCs are implicated in contributing to resistance against cisplatin and paclitaxel, both in vitro and in vivo [128,129]. In the case of colorectal cancer, CSCs are thought to be key players in mediating resistance to a variety of chemotherapeutic agents [130–133]. Additionally, in cancers such as ovarian, pancreatic, prostate, and small-cell lung cancer, CSCs play a crucial role in resistance to chemotherapeutics [125]. CSCs contribute to chemoresistance using different mechanisms. These mechanisms include EMT, MDR or detoxification proteins, dormancy, tumor environment, self-renewal, epigenetic modification, and resistance to cell death caused by DNA damage [110].

Studies indicated that circulating tumor cells from patients with metastases co-express both EMT and stem cell markers [134]. Additionally, EMT induction or EMT transcription factor activation results in cancer cells acquiring stem-like characteristics [135]. Mani et al*.* [136] initially indicated that triggering EMT in immortalized human mammary epithelial cells (HMLEs) generated stem cell-like cells that had a CD44^high^/CD24^low^ expression pattern, which matches the antigenic profile typical of both human breast CSCs and normal mammary epithelial stem cells. It is significant that these cells also had other CSC characteristics, including self-renewal and an increased capability to form mammospheres, which is a characteristic trait of mammary epithelial stem cells. Moreover, they discovered that stem cell-like cells isolated from HMLE cultures had markers identical to those of HMLEs that underwent an EMT and had mammosphere formation ability. Furthermore, they indicated that stem-like cells extracted from mouse or human mammary glands, as well as mammary tumors, exhibit EMT markers. Remarkably, the transcription factor ZEB1, an EMT regulator, has a critical role in regulating stemness and developing chemoresistance in CSCs of malignant glioma. ZEB1 promotes chemoresistance by regulating the transcription of O-6-methylguanine DNA methyltransferase (MGMT) via miR-200c and C-MYB. Also, ZEB1 expression is correlated with reduced survival and poor temozolomide response in glioblastoma patients [137]. A recent study on esophageal cancer cells indicated that drug-resistant esophageal cancer cells had stemness characteristics and stem cell biomarkers and were prone to EMT. Additionally, drug-resistant cells displayed decreased expression of the epithelial protein biomarkers Claudin-1, ZO-1, and E-cadherin and increased expression of the mesenchymal protein biomarkers vimentin, N-cadherin, and the transcription factor β-catenin [138]. Consequently, EMT promotes cancer cells to acquire stem cell-like features, which enhance cell invasion and drug resistance [139]. Various signaling pathways, including the Hedgehog, Notch, Wnt, platelet-derived growth factor (PDGF), and NF-κB signaling pathways, as well as miRNAs, have been recommended to participate in this procedure [65,68,140–142]. In TNBC cells, the sensitivity to cisplatin and doxorubicin was increased by silencing of Notch1, which suppressed the AKT pathway and led to a decrease in EMT [143]. Recently, a variety of nanomedicine methods for CSC-related treatment and diagnostics have been established [144,145]. For instance, targeting CSCs using multifunctional magnetic nanoparticles through a combination of chemotherapy and hyperthermia has proven to be an effective cancer treatment strategy [146]. The in vivo study exhibited that the miR-125b-5p nanomedicine that targets EMT and CSCs has successfully inhibited tumors [146]. Another nanomedicine approach involves utilizing co-loaded liposomes containing cabazitaxel and the CSC inhibitor silibinin to specifically target CD44 receptors on CSCs [147].

#### miRNAs

**Fig. 4.**
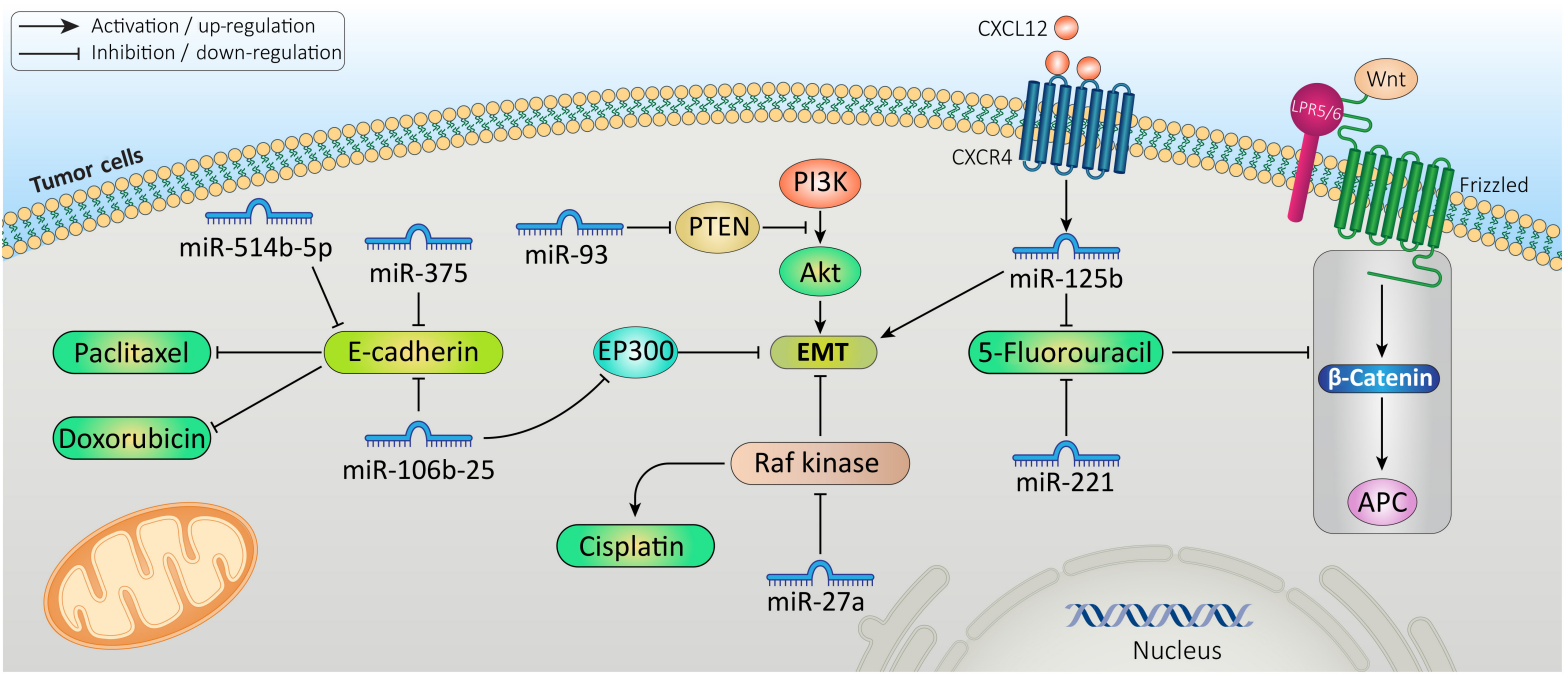
miRNAs cause EMT-induced drug resistance. miRNAs may promote chemoresistance by directly targeting epithelial markers. Different miRNAs by affecting E-cadherin, PTEN, RAF kinase, and Wnt/β-catenin pathway can affect drug resistance driven by EMT.

Multiple miRNAs are indicated to co-regulate drug resistance and EMT [148]. Through direct targeting of epithelial markers, miRNAs can contribute to chemoresistance (Fig. 4) [149]. For instance, in cervical cancer, miR-375 directly suppresses E-cadherin, which results in paclitaxel chemoresistance [150]. In colorectal cancer, miR-514b-5p has pro-metastatic properties by down-regulating E-cadherin expression, thereby promoting drug resistance. Interestingly, miR-514b-3p, transcribed from the same RNA hairpin, acts in a contrasting manner by reversing EMT-associated drug resistance. It enhances the expression of epithelial markers while reducing mesenchymal marker levels, leading to decreased cell migration, invasion, and resistance to drugs [151]. In breast cancer, the miR-106b-25 cluster induces doxorubicin resistance by suppressing EP300, a transcriptional activator of E-cadherin. EP300 suppression promotes cells with the phenotype characteristics of cells undergoing EMT, including increased cell motility and invasion and the capacity to proliferate following doxorubicin treatment [152]. One of the most important signaling pathways in controlling EMT-associated chemoresistance is the Wnt pathway. By targeting on the APC/Wnt/β-catenin pathway, miR-125b induced the EMT process and 5-fluorouracil resistance in colorectal cancer. Besides, CXCL12/CXCR4 could up-regulate miR-125b expression [153]. Furthermore, in esophageal cancer, miR-221 promoted 5-fluorouracil resistance through the Wnt/β-catenin pathway by directly targeting Dickkopf-2 expression [154]. Moreover, the tumor suppressor PTEN could inhibit EMT by suppressing the phosphatidylinositol 3-kinase (PI3K)/AKT signal. In breast cancer, miR-93 contributes to inducing EMT and doxorubicin resistance through PTEN suppression [155]. In lung adenocarcinoma, miR-27a was significantly up-regulated in cisplatin-resistant A549/CDDP cells. miR-27a could suppress Raf kinase inhibitory protein, which results in EMT induction and cisplatin resistance [156].

## EMT and Drug Resistance in HCC

The transition in cell phenotype is a crucial component leading to the development of chemoresistance in HCC. Recently, growing interest has been focused on the function of EMT in HCC progression, drug resistance development, and HCC treatment [157,158]. EMT causes differentiated HCC cells to revert to an undifferentiated or stem cell-like state, promoting metastasis and drug resistance [158].

Compared to well-differentiated liver cancer cell lines (Hep3B, HepG2, and Huh-7), poorly differentiated cell lines (HLE, HLF, and SK-Hep1) exhibit a loss of E-cadherin, express mesenchymal markers like N-cadherin, and demonstrate increased invasiveness as well as resistance to sorafenib, cisplatin, and doxorubicin. Additionally, according to clinical observations, poorly differentiated HCC is more resistant to chemotherapy and has a poor prognosis [159,160]. Moreover, EMT in HCC tumors is spatially heterogeneous, with diverse phenotypes and regulatory mechanisms in perivascular versus hypoxic niches. Blood vessel-rich perivascular regions stimulate EMT, which is characterized by increased expression of twist and vimentin and encourages tumor cell intravasation and dispersion. Hypoxic tumor areas increase tumor aggressiveness, stemness, and resistance to treatment by inducing EMT via HIF-1α and associated pathways. In HCC, this spatial heterogeneity affects tumor growth and metastatic patterns. Understanding this heterogeneity is critical for generating targeted treatments that incorporate the tumor microenvironment’s spatial context to effectively block EMT-driven metastasis and therapy resistance [23,161–163]. The following section discusses the potential molecules or pathways responsible for EMT-induced drug resistance in HCC.

### TGF-β signaling pathway

**Fig. 5.**
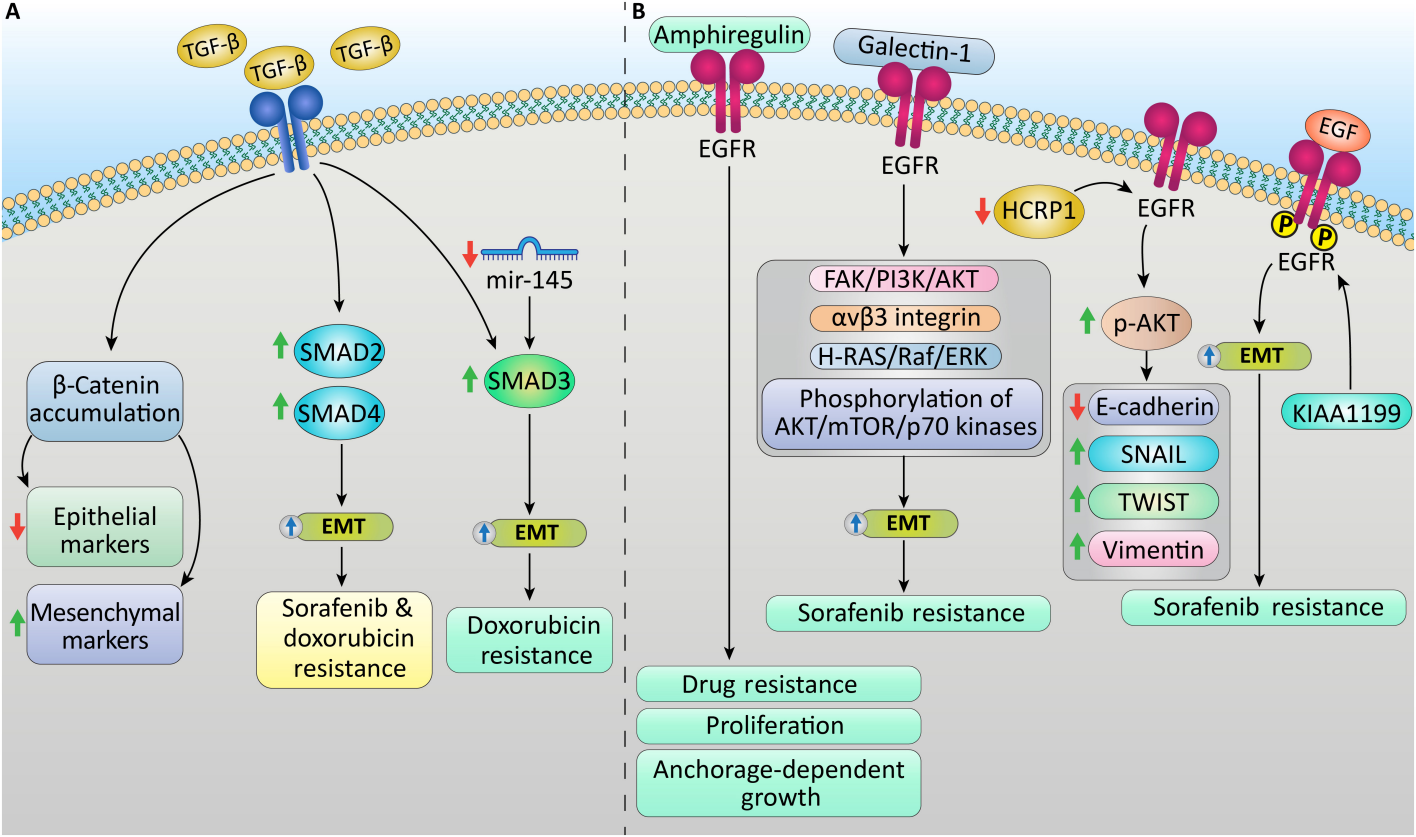
Signaling pathways responsible for EMT-induced drug resistance in HCC. (A) TGF-β signaling pathway induces EMT by prompting β-catenin accumulation in the nucleus and enhancing SMAD2, SMAD4, and SMAD3 expression, which results in sorafenib and doxorubicin resistance in HCC. (B) The EGFR signaling pathway induces drug resistance in HCC by affecting the EMT mechanism through the FAK/PI3K/AKT and H-Ras/Raf/ERK pathways and increasing the phosphorylation of AKT, mTOR, and p70 kinases.

One of the most important regulatory factors in the liver cancer microenvironment is TGF-β, which also has a significant regulatory role in the EMT. In addition, there is a strong correlation between the liver cancer’s multidrug resistance and TGF-β’s regulatory impact on the tumor microenvironment [164]. Induction of EMT in HCC is strongly influenced by the activation of the TGF-β signaling pathway (Fig. 5A) [165]. TGF-β promotes β-catenin accumulation in the nucleus, resulting in reduced epithelial marker expression and an up-regulation of stemness-related markers [166]. Elevated plasma levels of TGF-β1 have been linked to a poor therapeutic response to sorafenib and regorafenib in patients with advanced HCC [167,168]. The relationship between activation of the TGF-β pathway in HCC, increasing EMT, and promoting sorafenib resistance has been discovered [169,170]. SMAD proteins are components of the TGF-β pathway. In HepG2 and HuH7 cells, down-regulation of miR-145 resulted in doxorubicin resistance by enhancing SMAD3 expression. Additionally, up-regulation of miR-145 suppressed SMAD3-related EMT features, resulting in higher E-cadherin expression and lower vimentin levels [171]. Zhou et al*.* [169] found that overexpression of SMAD2 and SMAD4 was associated with enhanced EMT, which in turn caused a mesenchymal phenotype and increased resistance to doxorubicin and sorafenib in both HCC patients and in vitro model. Furthermore, down-regulation of miR-125b, a miRNA whose expression is significantly reduced in HCC, has been connected to the development of chemoresistance in HCC cells. Shrestha et al*.* [172] discovered that PD-L1 silencing and TGF-β1-induced EMT inhibition together re-sensitize HCC cells to sorafenib. According to a recent study by Modi et al. [173], sorafenib, an inhibitor of RAF kinase and VEGFR-2, can counteract the EMT induced by TGF-β in HepG2 cells by up-regulating E-cadherin and down-regulating vimentin and SNAIL. VEGFR-2 inhibitors may thus be effective against malignant cells with mesenchymal characteristics. According to recent research, autophagy could significantly induce drug resistance in HCC cells through molecular pathways such as TGF-β, NF-κB, Beclin 1, p62, NRF2, MAPK, and noncoding RNAs [174,175]. Moreover, EMT triggered by TGF-β stimulates PI3K/AKT signaling, a downstream pathway that LCSC markers like CD133 employ to control P-gp expression. This convergence on PI3K/AKT promotes drug efflux and resistance by increasing P-gp transcription and activity [176,177].

### EGFR signaling pathways

The EMT condition influences the responsiveness of HCC cells to therapies targeting the epidermal growth factor receptor (EGFR). Fuchs et al*.* [178] analyzed a set of 12 HCC cell lines, categorizing them as epithelial or mesenchymal based on their levels of E-cadherin and vimentin. Their findings revealed that mesenchymal cell lines exhibited greater resistance to EGFR inhibitors compared to epithelial lines, alongside higher expression of AKT and signal transducer and activator of transcription 3 (STAT3) and increased integrin-linked kinase levels. Amphiregulin, an EGFR ligand that does not appear in normal livers, is up-regulated in most cases of liver cancer due to chronic liver injury. Medication resistance, in vivo tumorigenic possibility, anchorage-independent growth, and proliferation rate were all elevated in SK-Hep1 cells overexpressing amphiregulin. [179]. Galectin-1 (Gal-1) is another signal that could promote EMT by altering EGFR pathways in HCC cells. In HCC cells, dysregulation of Gal-1 expression overactivated the FAK/PI3K/AKT and H-Ras/Raf/extracellular signal-regulated kinase (ERK) pathways and increased the phosphorylation of AKT, mTOR, and p70 kinases, as well as the expression of the αvβ3 integrin. These pathways’ dysregulation resulted in the EMT induction and increased sorafenib resistance. Furthermore, Gal-1 overexpression in tumors is correlated with a decreased response to sorafenib and poor HCC survivability outcomes [180]. Xu et al*.* [181] reported that down-regulation of hepatocellular carcinoma-related protein 1 (HCRP1) induces EGFR activation and EMT, which in turn enhances HCC cell migration and invasion. HCRP1 down-regulation enhanced the EMT phenotype in HepG2 cells through increased Snail and Twist1 and activation of AKT phosphorylation. Recent studies indicated that KIAA1199 activates the EGF/EGFR-dependent EMT process, which results in sorafenib tolerance and the metastasis of HCC (Fig. 5B) [182]. According to Jin et al. [183], HCC cells become more sensitive to lenvatinib when EGFR is inhibited. The combination of the EGFR inhibitor gefitinib and lenvatinib has strong anti-proliferative effects in xenografted liver cancer cells, immunocompetent animal models, patient-derived HCC tumors in mouse models, and liver cancer cell lines that express EGFR. Lenvatinib with gefitinib produced significant clinical responses in 12 patients with advanced HCC who had not responded to lenvatinib treatment. Following 4 to 8 weeks of combination treatment, patients with HCC tumors with high EGFR expression showed a partial response.

### Liver cancer stem cells

According to the CSC model, a subpopulation of tumor stem cells in cancer drives tumor growth. This model explains a number of clinical observations in HCC (as well as other cancers), such as the inevitable recurrence of tumors following successful first chemotherapy and/or radiation therapy, the condition of tumor dormancy, and treatment resistance [184].

The EMT process helps to create and sustain the CSC population in a number of cancers, such as HCC, which results in immunological evasion and treatment resistance [185]. Liver cancer stem cells (LCSCs) can come from two sources: (a) mature hepatocytes that undergo phenotypic reprogramming and dedifferentiation as a result of an inflammatory microenvironment and mutation buildup during carcinogenesis or (b) liver stem cells or progenitor cells by obtaining the oncogenic mutations that counteract the usual proliferation limits found in healthy stem cells [186,187]. LCSCs and cells that undergo EMT enhance the heterogeneity of cells within the tumor and have similar genetic characteristics, including elevated expression of surface glycoproteins of cell adhesion (CD44, CD133, CD13, CD24, CD90, EpCAM, and N-cadherin), keratin 19, aldehyde dehydrogenase 1A1, and transcription factors (SNAI1, SLUG, TWIST1, ZEB1, and ZEB2) [188–190]. High expression of the CSC markers CD133 and CD90 is correlated with a poorer answer to sorafenib in patients with HCC [191]. Furthermore, a mesenchymal-like phenotype and CD44 expression, associated with TGF-β pathway activation, are indicators of poor responsiveness to sorafenib in HCC cells. Fernando et al*.* [165]’s study on HCC cells in vitro and in vivo showed that mesenchymal-like phenotypic cells with high CD44 expression levels were resistant to the cell death caused by sorafenib. However epithelial-like cells were more susceptible to cell death induced by sorafenib. High expression of CD44 and CD133 leads to overexpression of ABC transporters in HCC cells [192,193]. The increased expression of ABC superfamily transporters, such as ABCB1, ABCC1, and ABCG2, renders CD133^+^ CD44^+^ HCC cells more resistant to chemotherapy drugs [192]. EpCAM, related to stemness gene expression, and α-fetoprotein (AFP) expression, have been suggested as indicators for various HCC phenotypic subgroups. In particular, the EpCAM^+^/AFP^+^ (hepatic stem cell-like) and EpCAM^−^/AFP^+^ (hepatocytic progenitor-like) groups have been related to induced drug resistance. This is because of improved cell survival, mostly caused by overactivation of the Wnt/β-catenin pathway [194]. Furthermore, sorafenib exposure enriched EpCAM^+^ cells in HCC patient-derived cells, which could facilitate acquired sorafenib resistance development [195]. Recently, Tiwari et al*.* [196] reported that piperine has antiproliferative activity against CD44^+^/CD133^+^ CSCs derived from HepG2 cells. Piperine also induces cell cycle arrest at the G1/G0 phase, which impairs cell cycle progression. Investigating the effect of piperine against the EMT induced by TGF-β in hepatocarcinogenesis showed that piperine was found to be able to repress the epithelial marker (E-cadherin). However, it was unable to down-regulate the levels of vimentin and SNAIL. Shrestha et al*.* [185] discovered that CSCs derived from human HCC have mesenchymal characteristics with elevated expression of immune checkpoints. This study indicated that the combination treatment strategy using SB431542 to block TGF-β1-induced EMT, coupled with immune checkpoint inhibition through PD-L1 and CD73 knockdown alongside sorafenib, could effectively target an invasive, drug-resistant subgroup of CSCs derived from HCC. It is commonly acknowledged that CSCs have a substantial role in both acquired and primary drug resistance. In order to overcome medication resistance and enhance the therapeutic success in HCC, hepatic CSC-targeted therapy is thought to be a potential approach in future applications [197]. Targeting LCSC in EMT-induced drug resistance has several limitations. There is mounting evidence that tumor cells with stem cell-like traits are more resistant to traditional treatment methods than those without stem cell traits. The characteristics of CSCs, such as their plasticity, quiescence, CSC niches, and enhanced drug efflux activity, are closely related to the processes by which resistance develops and limitations of treatment [198].

### Noncoding RNAs

**Fig. 6.**
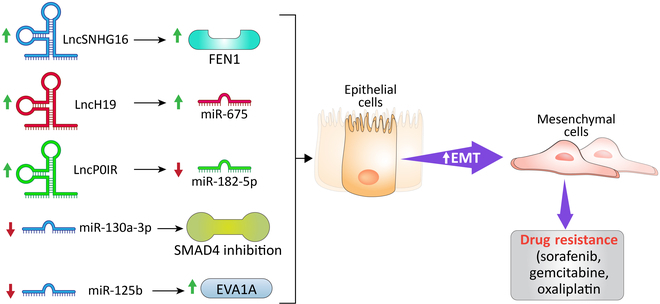
Noncoding RNA-induced drug resistance in HCC. Several noncoding RNAs, including long noncoding RNAs (LncSNHG16, lncH19, and lncPOIR) and microRNAs (miR-130a-3p and miR-125b), contribute an essential part in drug resistance in HCC.

Several studies have shown that noncoding RNAs (ncRNAs), such as miRNAs, long noncoding RNAs (lncRNAs), and circular RNAs (circRNAs), are key contributors to drug resistance in HCC [199]. Some ncRNAs are involved in drug resistance through EMT modulation [186,200]. Therefore, growing number of studies have supported the importance of interplay between the ncRNAs and EMT-associated resistance in HCC [201,202]. Liu et al*.* [203] reported that miR-130a-3p regulates gemcitabine resistance. miR-130a-3p was down-regulated in gemcitabine-resistant HCC (GR-HCC) cells, and miR-130a-3p overexpression inhibited cell migration and invasion. Additionally, it has been demonstrated that miR-130a-3p regulates EMT and cell invasion through Smad4 inhibition in GR-HCC cells. MiR-125b has been shown to counteract oxaliplatin resistance in HCC by down-regulating EVA1A, which in turn inhibits both autophagy and EMT processes. Analysis of oxaliplatin-sensitive and oxaliplatin-resistant HCC cell lines revealed that miR-125b expression was lower in the resistant cells. Furthermore, by preventing cell division, migration, and EMT, miR-125b overexpression in susceptible cells reduced resistance to oxaliplatin. Furthermore, cyclin D1 and N-cadherin’s down-regulations and E-cadherin elevation at both mRNA and protein levels are caused by miR-125b overexpression in resistant cells, suggesting that it suppresses EMT [204]. Provvisiero et al. [205] revealed that vitamin D can restore HCC cells resistant to the mTOR inhibitor everolimus by increasing the miR-375 expression and therefore decreasing the expression of multiple oncogenes involved in EMT. Additionally, c-MYC has been identified as a novel target of miR-375. These findings might provide a novel method to overcome mTOR inhibitor resistance in HCC treatment. LncSNHG16 expression is markedly elevated in HCC cells and is particularly linked to HCC invasiveness and poor patient outcomes. SNHG16 can act as an endogenous sponge for miR-140-5p and increase flap endonuclease 1 (FeN1), an oncogene associated with many cancers. It has been demonstrated that FeN1 silencing inhibits EMT, which prevents progression and metastasis in HCC. In conclusion, SNHG16 affects the EMT of HCC cells by impacting the miR-140-5p/FeN1 axis, which leads HCC cells to become resistant to sorafenib [206–208]. In HCC cells, lncH19 expression correlates negatively with sorafenib sensitivity. LncH19 knockdown can increase the susceptibility of HCC cells to sorafenib by suppressing EMT. Remarkably, H19 can increase miR-675 expression to induce EMT [209]. In addition to H19, it has also been demonstrated that other lncRNAs, such as LINC01089 and CYTOR, can target the EMT process and impact malignant features like invasion, metastasis, and proliferation, which affect cancer drug resistance [210–213]. According to Sun et al*.* [213], lncLIMT (LINC01089), which suppresses EMT and miR-665 expression, reduces sorafenib resistance and slows down tumor development in nude mouse models. Moreover, lncPOIR suppresses sorafenib sensitivity and enhances HCC development by acting as a sponge for miR-182-5p, inhibiting the miR-182-5p expression, and inducing EMT. LncPOIR knockdown reverses the EMT and sensitizes HCC cells to sorafenib (Fig. 6) [214]. According to Hirao *et.al*., miR-125b-5p is up-regulated in HCC cell lines, which are resistant to sorafenib, and its overexpression inhibits the expression of ataxin 1 (*ATXN1*), which causes EMT. Accordingly, miR-125b-5p has been shown to increase sorafenib resistance in vivo models as well [215]. Besides, there are still certain issues with miRNA-based treatments, namely, the off-target effect and the absence of an ideal delivery mechanism. The in vivo delivery of miRNAs remains a difficulty due to their fast excretion, inappropriate intracellular release, poor biostability, endosomal escape, and immunogenicity. Additionally, stable therapeutic targeting is difficult since EMT is a dynamic and reversible process, and miRNAs implicated in EMT regulation may have diverse effects based on the tumor microenvironment and cancer stage. Moreover, certain miRNAs have dual functions in drug resistance and EMT. Therapeutic targeting is made more difficult by this contradictions [20,148].

## Further Innovative Strategies and Future Perspectives

Targeting EMT has been shown in numerous research projects to aid in overcoming chemoresistance; nanotechnology and nanomedicine are emerging technologies as potential approaches to accomplish this goal. For instance, some studies address the possibility of using nanotechnology to prevent EMT for treating chemoresistant solid tumors, such as breast cancer, lung cancer, pancreatic cancer, glioblastoma, ovarian cancer, gastric cancer, and HCC [216]. Additionally, novel therapeutic targets for overcoming drug resistance are posttranslational EMT regulators including Hakin-1, FBXW7, and USP27X [20,217–219]. Additionally, recent research uses pharmacogenomics and bioinformatics to evaluate how EMT affects therapy resistance and to create new pharmacological approaches for the future. For example, according to RACIPE mathematical modeling, Twist1 and E-cadherin have a significant negative correlation, and Twist1 and vimentin have a positive correlation. Also, in the context of EMT, Twist1 overexpression increases genomic instability, which leads to cellular heterogeneity and potentially chemoresistance [220]. A variety of recent studies in different types of cancer have revealed a correlation between EMT and the regulation of certain ribosomal proteins. Certain ribosomal proteins could regulate cell migration and modify EMT, ultimately leading to chemoresistance. For instance, down-regulation of the ribosomal protein uL3 contributes to enhanced cell migration and an EMT that leads to chemoresistance [221–224]. Another new study about protein arginine methyltransferases (PRMTs) suggests that they enhance the development and maintenance of drug-tolerant cells via numerous methods, which includes EMT. PRMTs are the enzymes in charge of arginine epigenetic methylation, which controls a number of physiological and pathological processes. Therefore, PRMTs make appealing therapeutic targets for overcoming drug resistance to anti-cancer medications [225]. Additionally, the possibility of overcoming drug resistance has been explored for novel medications that target epigenetic regulators and pharmacological combinations that target multiple resistance pathways. Finding the biomarkers for medication resistance can help with the generation of customized precision treatment [13]. Based on another novel study, the tremendous potential for cuproptosis—a recently discovered kind of cell death caused by copper—in cancer research communities has sparked a great deal of interest. Copper-based treatments hold promises for addressing chemotherapy-resistant malignancies and may help to inhibit tumor growth. This study suggests that targeting cuproptosis could serve as a potential anticancer strategy or an effective approach to overcome drug resistance in cancer [226]. Furthermore, combining immunotherapy and chemotherapy can be a successful strategy to overcome drug resistance. For instance, a clinical trial for unresectable HCC patients has shown that combination therapy of atezolizumab (anti-PD-L1 Ab) and bevacizumab (anti-VEGF Ab) provided significantly better overall survival rate and progression-free survival outcomes than sorafenib [227]. Another recent clinical trial, HIMALAYA, a phase 3 randomized trial, showed promising outcomes for patients with unresectable HCC treated with a combination of durvalumab (anti-PD-L1 Ab) and tremelimumab (anti-CTLA4 Ab) [228]. These new studies open novel pathways for treating EMT-induced drug resistance in HCC and other similar cancers. Therefore, studying the reasons for EMT-induced drug resistance and solutions is so important and could help to establish new therapeutic protocols.

## Conclusion

One of the main challenges in treating HCC is overcoming drug resistance. Due to primary and acquired drug resistance, a significant number of patients with advanced HCC fail to achieve long-term improvement with systemic therapy, and this, collectively with the disease’s growing incidence, makes HCC a very lethal cancer. Numerous studies showed that EMT contributes significantly to drug resistance. This process is modulated by the regulation of several signaling pathways, including NF-κB, Wnt, Hedgehog, Notch, TGF-β, AKT, and miRNAs. New treatment approaches that target EMT in order to restore drug sensitivity in HCC should be developed in light of our growing understanding of EMT and drug resistance. The combination of bioinformatics, pharmacogenomics, and chemical genomic data will be essential to find novel chemosensitizing medications and therapeutic targets that can overcome resistance to several chemotherapies. Further studies in this field could help develop novel therapeutic strategies that might significantly enhance the treatment of patients with advanced HCC. Developing research on EMT could considerably influence the future generation of cancer therapeutics for drug resistance.

## Data Availability

All data supporting the findings of this study are available within the article or from the corresponding author upon reasonable request.
